# Population genetic structure of *Aedes polynesiensis *in the Society Islands of French Polynesia: implications for control using a *Wolbachia-*based autocidal strategy

**DOI:** 10.1186/1756-3305-5-80

**Published:** 2012-04-24

**Authors:** Corey L Brelsfoard, Stephen L Dobson

**Affiliations:** 1 Department of Entomology, S-225 Ag. Science Center North, University of Kentucky, Lexington, KY 40546, USA

**Keywords:** *Aedes polynesiensis*, Genetic structure, French Polynesia

## Abstract

**Background:**

*Aedes polynesiensis *is the primary vector of *Wuchereria bancrofti *in the South Pacific and an important vector of dengue virus. An improved understanding of the mosquito population genetics is needed for insight into the population dynamics and dispersal, which can aid in understanding the epidemiology of disease transmission and control of the vector. In light of the potential release of a *Wolbachia *infected strain for vector control, our objectives were to investigate the microgeographical and temporal population genetic structure of *A. polynesiensis *within the Society Islands of French Polynesia, and to compare the genetic background of a laboratory strain intended for release into its population of origin.

**Methods:**

A panel of eight microsatellite loci were used to genotype *A. polynesiensis *samples collected in French Polynesia from 2005-2008 and introgressed *A. polynesiensis *and *Aedes riversi *laboratory strains. Examination of genetic differentiation was performed using *F*-statistics, STRUCTURE, and an AMOVA. BAYESASS was used to estimate direction and rates of mosquito movement.

**Results:**

*F*_ST _values, AMOVA, and STRUCTURE analyses suggest low levels of intra-island differentiation from multiple collection sites on Tahiti, Raiatea, and Maupiti. Significant pair-wise *F*_ST _values translate to relatively minor levels of inter-island genetic differentiation between more isolated islands and little differentiation between islands with greater commercial traffic (i.e., Tahiti, Raiatea, and Moorea). STRUCTURE analyses also indicate two population groups across the Society Islands, and the genetic makeup of *Wolbachia *infected strains intended for release is similar to that of wild-type populations from its island of origin, and unlike that of *A. riversi*.

**Conclusions:**

The observed panmictic population on Tahiti, Raiatea, and Moorea is consistent with hypothesized gene flow occurring between islands that have relatively high levels of air and maritime traffic, compared to that of the more isolated Maupiti and Tahaa. Gene flow and potential mosquito movement is discussed in relation to trials of applied autocidal strategies.

## Background

*Aedes polynesiensis *is a day biting pest and the major vector of *Wuchereria bancrofti *and a secondary vector of Dengue virus in the South Pacific [1]. *A. polynesiensis *established concurrent with the arrival of man in the South Pacific, approximately 1500-3000 years ago and has spread throughout French Polynesia and other island groups ranging from Fiji to the Tuamotu Archipelago [2]. *A. polynesiensis *is adapted to ovipositing in both man-made (e.g., rain water catch basins, discarded bottles, buckets, and cans) and natural containers [3,4]. Natural containers that *A. polynesiensis *oviposits in include: coconut shells, rock holes, tree-holes, and crab holes generated by the gecardinid crab, *Cardisoma carnifex *(Herbst). The ability of *A. polynesiensis *to survive in numerous larval habitats has contributed to its widespread dispersal and the difficulty of control [2].

Attempts to control *A. polynesiensis *have been largely unsuccessful, despite the use of insecticides, biological control, and larval habitat removal [1,4-6]. Area-wide elimination techniques such as the sterile insect technique and other autocidal strategies are being developed as alternative control measures [7]. Understanding the population structure of *A. polynesiensis *in French Polynesia is important for defining the scale on which vector control using area-wide techniques is likely to be most effective. More specifically, characterization of the population genetic structure will help to define the level of population structuring, including the potential for cryptic subgroups that may be unaffected by releases of incompatible males [8]. An improved understanding of gene flow can help to design applied strategies that are less susceptible to mosquito movement.

A *Wolbachia*-based incompatible insect technique (IIT) analogous to the sterile insect technique (SIT) has been suggested as an alternative for control of *A. polynesiensis *[7]. In the *Wolbachia*-based IIT strategy, female sterility is artificially sustained by repeated inundative releases of incompatible males, resulting in mosquito population decrease and possibly elimination. A strain (CP) that harbors an incompatible *Wolbachia *infection has previously been developed for a potential release for field-testing of a *Wolbachia*-based IIT strategy in French Polynesia [7]. The CP strain has shown high rates of CI and males have been shown to be competitive for mates with the indigenous natural population [7,9,10]. As previously described, the CP strain was generated through a series of interspecific hybridizations with *A. riversi *(AR) to transfer the maternally inherited endosymbiotic *Wolbachia *infection from *A. riversi *into the *A. polynesiensis *genetic background [7]. *A. polynesiensis *collected from Maupiti served as the *A. polynesiensis *genotype donor. As a result of the introgression of the *A. polynesiensis *genetic background, it is important to ensure the genetic makeup of the CP strain is similar to the indigenous population and different from that of *A. riversi *prior to potential releases.

Previous population genetic studies of *A. polynesiensis *using isoenzymes have shown genetic differentiation between populations on islands from different archipelagos of French Polynesia [11]. A more recent study using microsatellite markers and rDNA ITS2 (internal transcribed spacer 2) sequences, suggested that significant genetic differentiation exists and limited gene flow occurs between Moorea and Fiji [12]. Polynesian islands are categorized into either 'high islands' with volcanic mountains and rain forests or 'low islands' consisting of coral atolls a few meters above sea level. Due to the different ecology of the islands and the geographic isolation of some islands it has been suggested that there may be cryptic species of *A. polynesiensis *associated with different ecotypes, however, previous studies using isoenzymes have suggested that there is no genetic differentiation associated with habitat differences [13].

The goal of the present study was to better understand the population genetic structure of *A. polynesiensis *on a microgeographical scale, using samples collected from five islands in the Society Islands of French Polynesia. Vector population structure and movement patterns are relevant to the ecology and evolution of *A. polynesiensis*, gene flow and mosquito movement [14], insecticide susceptibility [15], disease epidemiology and control [16], previously observed variation between populations in their vector competency [17,18], and autocidal control strategies that are under development [7,19]. Furthermore, we compare the genetic makeup of a laboratory strain intended for release as part of a *Wolbachia-*based population suppression strategy, to that of the indigenous population and *A. riversi*. Results are discussed in reference to the development of area-wide vector control efforts to control *A. polynesiensis*.

## Methods

### Mosquito collections and DNA extractions

**Figure 1:**
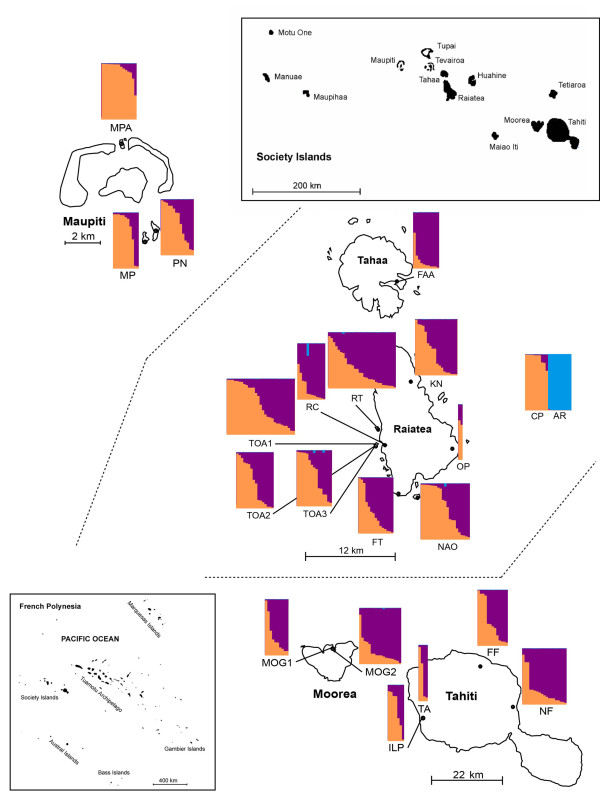
**Bayesian STRUCTURE analysis of *A. polynesiensis *collected in the Society Islands of French Polynesia, 2005-2008, the CP strain (laboratory strain harboring an incompatible *Wolbachia *infection and *A. polynesiensis *genetic background), and *Aedes riversi *(AR)**. Individual multilocus genotypes are plotted as vertical lines, in which the colored segments represent the membership coefficients for each of the three clusters. Individuals were re-ordered according to membership coefficient to each of the three clusters. Collection site location labels are below each STRUCTURE plot for each collection locality.

Adult mosquitoes were collected in 2005, 2007, and 2008. Collection locations are denoted in Figure 1 and Table 1. Field collected mosquito samples were stored in 95% EtOH until DNA extraction. DNA was extracted from ten adults from each of the laboratory strains: CP (incompatible hybrid strain) [7] and AR (*A. riversi*) [20], which have been in a laboratory colonies for > 30 generations. For DNA extraction, whole mosquitoes were washed with sterile diH_2_O and homogenized using a mini-bead beater (BioSpec Products, Inc., Bartlesville, OK) in a 1.5 ml eppendorf tube containing 100 μl of sterile diH_2_O and a 2.5 mm glass bead (BioSpec Products, Inc., Bartlesville, OK). The homogenate was then used for extraction with a DNeasy tissue kit (Qiagen, Valencia, CA), following manufacturer's instructions.

**Table 1:** *A. polynesiensis *populations from the Society Islands of French Polynesia

Island	Collection site (abbreviation)	Collection date	*n*	Coordinates
Maupiti	motu Pitiahe (MP)	November 2005	11	S 16° 28.8', W 152° 14.9'
Maupiti	motu Tiapaa (PN)	November 2005	14	S 16° 28.5', W 152° 14.5'
Maupiti	motu Paeso (MPA)	November 2005	15	S 16° 25.3', W 152° 15.6'
Tahaa	Faaha Bay (FAA)	March 2008	11	S 16° 37.7', W 151° 27.0'
Raiatea	motu Tiano (RT)	November 2005	29	S 16° 49.8', W 151° 29.3'
Raiatea	motu Toamaro (TOA1)	November 2005	29	S 16° 51.2', W 151° 29.3'
Raiatea	Commune (RC)	November 2005	12	S 16° 51.1', W 151° 29.4'
Raiatea	Fetuna (FT)	March 2008	15	S 16° 55.1', W 151° 26.9'
Raiatea	motu Nao Nao (NAO)	March 2008	21	S 16° 55.1', W 151° 25.9'
Raiatea	Kaoha Nui Ranch (KN)	March 2008	18	S 16° 46.6', W 151° 26.3'
Raiatea	Opoa (OP)	March 2008	2	S 16° 50.7', W 151° 22.9'
Raiatea	motu Toamaro (TOA2)	March 2008	15	S 16° 51.2', W 151° 29.3'
Raiatea	motu Toamaro TOA3)	July 2008	16	S 16° 51.2', W 151° 29.3'
Tahiti	Paea (TA)	February 2007	4	S 17° 43.9', W 149° 34.7'
Tahiti	Nui Faaoen (NF)	February 2008	19	S 17° 41.1', W 149° 18.3'
Tahiti	Faarumi Falls (FF)	February 2008	14	S 17° 32.1', W 149° 24.0'
Tahiti	Paea (ILP)	March 2008	7	S 17° 43.9', W 149° 34.7'
Moorea	Gump Station (MOG1)	November 2005	10	S 17° 29.4', W 149° 49.6'
Moorea	Gump Station (MOG2)	February 2008	18	S 17° 29.4', W 149° 49.6'
		Total	280	

### Microsatellite analysis

Ten microsatellite loci were used in this study. Six microsatellite loci for *A. polynesiensis *were characterized as part of a previous study (Table 2). Three loci developed previously for *A. polynesiensis *and one *Aedes albopictus *locus was characterized for *A. polynesiensis *as part of this study (Table 2). Repeat regions were located as previously described [21]. One primer of the pair for each locus was marked with WellRED dye (Integrated DNA technologies, Coralville, IA). Amplicons of the two most common alleles for the four uncharacterized loci were sequenced by Macrogen USA (Rockville, MD) to confirm expected repeat motif and amplification size. Polymerase chain reaction (PCR) was performed using a PT-100 thermal cycler (MJ research Inc., Waltham MA). PCRs were performed using Qiagen multiplex PCR kits, following manufacturers instructions (Qiagen, Valencia, CA). The thermocycler program used for amplification consisted of 94°C for 5 min, 33 cycles of 94°C for 25 sec, 50°C for 28 sec, 72°C for 30 sec, followed by an extension step of 72°C for 20 min. Amplifications were found to be sufficient for sizing in a Beckman Coulter 2500 automated DNA sequencer (Beckman Coulter, Fullerton, CA), using 0.5 μl of isolated DNA in a 12.5 μl reaction. Size standards and an amplicon from a sequenced allele were used as controls, to avoid the misidentification of alleles.

**Table 2:** Microsatellite primers used in this study

Locus	Primer sequence (5'-3')	Repeat	Cloned size (bp)	Reference
APT1	F: CTCTGGCCAAAACAGAGACC R: CAGCAGTTCAAAGGATGTCG	(GCC)_5_	164	This study
APT2	F: GATCCTTCGGGATGACACAG R: GAGCAAAAGTAGCCCACAGC	(GT)_11_	154	This study
APT6	F: TTCCTCGTTTCTCATTTTTCC R: GAGACCCAAATCAAAGACACG	(GT)_10_	181	This study
AealbA9	F: TGGGACAAGAGCTGAAGGAT R: CTCGTTCTCTACTCTCTCCGTT	(AC)_4 _N_4_(AC)_8 _N_4 _(AC)_2 _N_2 _(AC)N_2 _(AC)N (AC)N_2_(AC)	152	[22]
AP1	F: GCACCAGAGCAAAAGTAGCC R: GGGAAGAGAAAGAAGCACCC	(AC)_14 _N_3_(CG)_5_	129	[21]
AP2	F: ATTACCGCCGTACTGCTGAG R: CATCACCACCATCACCAAAC	(TGC)_12 _N_6_(TGA)_4_	148	[21]
AP3	F: AGGAGTGTTCTGCTGTTGGG R: GCAAACTTTTCCCTTCCTCC	(TGC)_5_	111	[21]
AP4	F: CCACAAAAAGCCAAAAGAGC R: ACTTGGGAGTGATGGTGTGG	(TCA)_6_	151	[21]
AP5	F: AGATGGTGCTGGGTGAAGAC R: AGTGCAAACAACACCAGCAG	(TGG)_4 _N_30_(TGC)_4_(TGT)_3 _N_12_(TGA)_3_	144	[21]
AP6	F: CTACTCTGTAGACCATGGCGG R: TCAGCGGAGAGTTGATGTCC	(CT)N_26_(CAC)_3 _N_36_(CAC)_3_(CAG)_4_(CA)_3_	186	[21]

### Genetic analysis

Microsatellite variability, *F*_IS_, and observed and expected heterozygosity for each locus was determined using FSTAT (v. 2.9.3; [23]). MICRO-CHECKER [24] was used to determine if null alleles were present in the data set. Each locus and population was tested for deviations from Hardy-Weinberg equilibrium (HWE) expectations with exact tests using GENEPOP (v. 3.4; [25]). GENEPOP was used to examine for linkage disequilibrium among all pairs of loci within each collection site. All GENEPOP analyses were performed using the following parameters: dememorization = 1,000, number of batches = 100, and number of iterations/batch = 1,000. To examine for population substructure within each island group, *F*_ST _values were calculated followed by overall tests for differentiation using a bootstrap corrected Fishers exact tests in FSTAT. To evaluate the level of differentiation between island groups, pair-wise *F*_ST _values [26] were calculated using FSTAT. To determine if null alleles were introducing bias into the analyses of differentiation, *F*_ST _values between populations were calculated using the ENA method implemented in FreeNA, which corrects for the presence of null alleles [27]. To determine if there was genetic differentiation between the main island of Raiatea and satellite islands (i.e., motu), main island samples were pooled and compared to each motu using pair-wise *F*_ST _values, as described previously. Pair-wise *F*_ST _values were calculated using FSTAT, to examine for temporal genetic variation between multiple year collections. The overall significance of *F*_ST _values for each island group was estimated by using bootstrap corrected Fishers exact tests in FSTAT. Bonferroni corrections were performed for all tests that involved multiple comparisons.

Bayesian analysis was implemented in the program STRUCTURE (v. 2.1; [28]), to infer population genetic structure from all samples collected. STRUCTURE uses multi-locus genotype data to determine the probability that an individual is derived from one of *K *hypothetical populations. Five independent runs were carried out for each value of *K *to check for consistency. To explore population genetic structure of all samples collected, *K *was allowed to vary from one to 12 using an admixture model. To examine for intra-island structure, *K *was allowed to vary for each island: Raiatea, one to nine, Maupiti, one to three, and Tahiti, one to four. For each analysis the burn-in length was set to 100,000 and the run length to 250,000 iterations. Real and simulated data have suggested that it is not straightforward to determine the optimal value of *K *when complex structure exists [29]. Hence, two methods for selecting *K *were used when examining the population genetic structure of all the islands sampled. The first method used was Δ*K *(a measure of the second order rate of change in the likelihood of *K*) [29]. The second method used posterior probabilities calculated by STRUCTURE. Clustering algorithms such as the ones used in STRUCTURE use unsupervised approaches that involve stochastic simulation. Thus, replicate cluster analyses may produce several different solutions for the estimated *K*. Differences are the result of 'label switching' of clusters across replicates and 'genuine multimodality'. CLUMPP [30] was used, which takes cluster membership coefficient matrices of multiple runs of STRUCTURE and permeates them so that all replicates are the closest match as possible. The CLUMPP analysis is based on a pre-defined *K *value and does not effect the determination of *K*. Subsequently, DISTRUCT [31] was used to display membership coefficients generated by CLUMPP. Two laboratory populations were also included in the STRUCTURE analysis (i.e., AR and CP).

To determine if geographic distance was influencing the observed pattern of genetic differentiation between main island groups, an isolation by distance analysis was conducted using Mantel tests within the sub-program Isolde (implemented in GENEPOP). Isolation by distance was tested by correlating linearized *F*_ST _values and the natural logarithm of the straight-line distance between islands.

As an additional analysis of genetic differentiation between island groups, a hierarchical analysis of population structure was performed, by estimating Nei's genetic distance values [32]. Nei's genetic distance values were calculated using GENDIST, available in the software package PHYLIP [33]. An un-rooted neighbor-joining tree was constructed using Nei's genetic distance values using NEIGHBOR, available in the software package PHYLIP [33]. DENDROSCOPE [34] was used to visually display the tree. BAYESASS (v.1.2; [35]) was used to estimate direction and rates of migration that has occurred more recently (i.e., within the last several generations) between different island groups. The island groups were composed of all collection sites pooled from each island. Samples were pooled from each island group to avoid sampling error, since many collection sites have a small sample size. The BAYESASS program simulation was run using 300,000 iterations, a burn in length of 999,999, and a sampling frequency of 2,000. BAYESASS defines migrants by hybrid genotypes and determines a migration rate *m*, which is defined as the proportion of first generation migrants.

## Results

### Microsatellite validation

Analyses of the ten-microsatellite loci show all to be polymorphic for at least one collection site (Table 3). Locus AP5 and AP6 were excluded from further analyses because of observed nominal polymorphism (Table 3). Sequencing confirmed that the two most common alleles from the uncharacterized loci were the same size as predicted by fragment analysis performed by the automated sequencer. HWE was tested for each locus in each population. Out of 152 probability tests performed for each locus, three tests were significant for locus AP4 after a Bonferroni correction (Table 3). In all cases, deviations from HWE were due to heterozygote deficiency. Among possible factors that might account for these deviations are inbreeding, the Wahlund effect, and/or null alleles. An analysis using MICRO-CHECKER, which checks for genotyping errors (e.g., non-amplified alleles, allelic dropout, and the scoring of stutter peaks) [24] detected the presence of null alleles in 14 out of 152 tests. To test if the presence of the null allele biased estimates of differentiation, pair-wise *F*_ST _values were calculated using the ENA null allele correction method [27]. Although there were some differences between the corrected and uncorrected estimates of genetic differentiation none were substantial, and no consistent bias was observed (Table 4). A global test of *F*_ST _also suggests little difference in *F*_ST _values when comparing all populations with and without the ENA correction (with = 0.060, without = 0.066).

**Table 3:** Microsatellite validation

		Locus									
		
Site		AP1	AP2	AP3	AP4	AP5	AP6	AealbA9	APT1	APT2	APT6
MP	N	11	11	11	11	11	11	11	11	11	11
	N_all_	3	3	1	2	1	1	7	2	4	2
	F_IS_	+0.433	-0.026	-	+0.259	-	-	+0.161	-0.053	+0.320	-0.053
	H_e_	0.63	0.18	-	0.48	-	-	0.65	0.17	0.65	0.17
	H_o_	0.36	0.18	-	0.36	-	-	0.55	0.18	0.45	0.18

MPA	N	15	15	15	15	15	15	15	15	15	15
	N_all_	5	3	2	2	1	2	4	2	3	2
	F_IS_	-0.405	-0.087	-0.273	+1	-	-	-0.018	-0.167	+0.152	-0.077
	H_e_	0.72	0.25	0.37	0.24	-	0.11	0.53	0.29	0.63	0.19
	H_o_	1	0.27	0.47	-	-	-	0.53	0.33	0.53	0.2

PN	N	14	14	14	14	14	14	14	14	14	14
	N_all_	5	2	3	2	1	1	4	3	3	2
	F_IS_	-0.283	+0.447	-0.490	+1*	-	-	-0.139	+0.218	+0.019	-
	H_e_	0.62	0.26	0.54	0.51	-	-	0.38	0.36	0.58	0.07
	H_o_	0.79	0.14	0.79	-	-	-	0.43	0.28	0.57	0.07

KN	N	18	18	18	18	18	18	18	18	18	18
	N_all_	5	4	2	2	1	2	8	2	5	2
	F_IS_	+0.058	-0.204	-	+1*	-	0.03	-0.018	+0.358	+0.220	-
	H_e_	0.65	0.56	0.06	0.49	-	0.15	0.82	0.52	0.49	0.06
	H_o_	0.61	0.67	0.06	**-**	-	0.06	0.94	0.33	0.39	0.05

FT	N	15	15	15	15	15	15	15	15	15	15
	N_all_	6	3	2	2	1	1	4	3	7	2
	F_IS_	-0.045	-0.479	-	+0.606	-	-	-0.067	-0.340	-0.273	-
	H_e_	0.64	0.64	-	0.33	-	-	0.25	0.45	0.69	0.07
	H_o_	0.67	0.93	0.06	0.13	-	-	0.27	0.6	0.87	0.06

OP	N	2	2	2	2	2	2	2	2	2	2
	N_all_	3	2	1	2	1	1	3	2	2	2
	F_IS_	-0.333	+1	-	+1	-	-	-0.333	-1	-	-
	H_e_	0.85	0.65	-	0.65	-	-	0.85	0.65	0.5	0.5
	H_o_	1	-	-	-	-	-	1	1	0.5	0.5

RC	N	12	12	12	12	12	12	12	12	12	12
	N_all_	6	3	4	4	2	1	5	2	3	1
	F_IS_	-0.186	+0.224	+0.102	+0.500	+1	-	+0.725	-0.132	-0.055	-
	H_e_	0.64	0.64	0.37	0.49	0.16	-	0.58	0.52	0.56	-
	H_o_	0.75	0.5	0.33	0.25	-	-	0.17	0.58	0.58	-

NAO	N	21	21	21	21	21	21	21	21	21	21
	N_all_	6	5	2	2	1	1	2	5	7	4
	F_IS_	-0.081	+0.077	-0.53	+0.834*	-	-	-	-0.255	+0.200	+0.152
	H_e_	0.7	0.62	0.14	0.56	-	-	0.05	0.57	0.65	0.33
	H_o_	0.76	0.57	0.14	0.1	-	-	0.05	0.71	0.52	0.29

RT	N	29	29	29	29	29	29	29	15	15	15
	N_all_	5	5	2	2	1	1	7	2	5	2
	F_IS_	-0.204	+0.058	+0.359	+0.208	-	-	+0.002	-0.167	+0.019	-0.037
	H_e_	0.58	0.59	0.16	0.39	-	-	0.55	0.52	0.61	0.13
	H_o_	0.69	0.56	0.1	0.31	-	-	0.55	0.6	0.6	0.13

TOA1	N	29	29	29	29	29	29	15	15	15	15
	N_all_	6	4	2	2	1	1	8	3	5	5
	F_IS_	-0.001	-0.090	-0.018	+0.125	-	-	-0.016	-0.181	-0.135	+0.576
	H_e_	0.66	0.57	0.07	0.36	-	-	0.59	0.4	0.71	0.31
	H_o_	0.66	0.62	0.07	0.31	-	-	0.6	0.47	0.8	0.13

TOA2	N	16	16	16	16	16	16	16	16	16	16
	N_all_	5	3	2	2	2	1	5	2	4	2
	F_IS_	-0.277	+0.540	-0.429	+1	-	-	+0.080	-0.050	-0.149	-
	H_e_	0.69	0.54	0.44	0.31	0.06	-	0.34	0.42	0.54	0.06
	H_o_	0.88	0.25	0.63	-	0.06	-	0.31	0.44	0.63	0.06

TOA3	N	15	15	15	15	15	15	15	15	15	15
	N_all_	5	3	1	3	1	1	4	2	3	4
	F_IS_	-0.102	-0.426	-	+0.691	-	-	+0.142	-0.359	-0.011	-0.094
	H_e_	0.73	0.52	-	0.42	-	-	0.62	0.5	0.66	0.31
	H_o_	0.8	0.73	-	0.13	-	-	0.53	0.67	0.67	0.33

FAA	N	11	11	11	11	11	11	11	11	11	11
	N_all_	5	2	1	2	1	1	6	2	3	1
	F_IS_	-0.165	+0.107	-	+0.643	-	-	+0.241	0	-0.026	-
	H_e_	0.47	0.51	-	0.25	-	-	0.71	0.45	0.18	-
	H_o_	0.55	0.46	-	0.05	-	-	0.55	0.45	0.18	-

MOG	N	10	10	10	10	10	10	10	9	9	9
	N_all_	4	4	2	2	1	1	5	2	4	2
	F_IS_	-0.000	+0.316	-0.200	+1	-	-	-0.248	-0.455	-0.347	-
	H_e_	0.6	0.43	0.34	0.19	-	-	0.57	0.47	0.68	0.11
	H_o_	0.6	0.3	0.4	-	-	-	0.7	0.67	0.89	0.11

MOG2	N	18	18	18	18	18	18	18	18	18	18
	N_all_	5	4	2	3	1	2	4	2	5	1
	F_IS_	-0.012	+0.163	-0.259	+0.785	-	-	+0.092	+0.329	+0.101	-
	H_e_	0.66	0.53	0.36	0.25	-	0.06	0.43	0.24	0.62	-
	H_o_	0.67	0.44	0.44	0.06	-	0.06	0.39	0.16	0.56	-

ILP	N	7	7	7	7	7	7	7	7	7	7
	N_all_	3	2	1	3	1	1	3	2	2	1
	F_IS_	+0.217	+0.478	-	+1	-	-	-0.200	+0.625	+0.368	-
	H_e_	0.54	0.53	-	0.61	-	-	0.49	0.36	0.44	-
	H_o_	0.43	0.29	-	-	-	-	0.57	0.14	0.29	-

TA	N	4	4	4	4	4	4	4	4	4	4
	N_all_	5	3	1	2	1	2	2	3	3	1
	F_IS_	+0.143	-0.091	-	-	-	-	+0.143	-0.600	-0.286	-
	H_e_	0.85	0.48	-	0.24	-	0.25	0.58	0.68	0.6	-
	H_o_	0.75	0.5	-	0.13	-	0.25	0.5	1	0.75	-

NF	N	19	19	19	19	19	19	19	19	19	19
	N_all_	4	6	1	2	1	2	2	2	4	1
	F_IS_	-0.093	-0.024	-	+0.780	-	-	-0.059	-0.029	-0.026	-
	He	0.63	0.62	-	0.24	-	0.05	0.15	0.1	0.56	-
	H_o_	0.68	0.63	-	0.05	-	0.05	0.16	0.1	0.57	-

FF	N	13	13	13	12	13	13	13	13	13	13
	N_all_	5	3	1	2	1	2	4	2	5	1
	F_IS_	+0.050	+0.177	-	+1	-	-	-0.043	-0.263	+0.072	-
	H_e_	0.65	0.55	-	0.39	-	0.08	0.22	0.37	0.66	-
	H_o_	0.62	0.46	-	-	-	0.08	0.23	0.46	0.61	-

Total	N	279	279	279	278	279	279	265	250	250	250
	N_all_	10	8	5	6	3	3	9	5	12	7
	F_IS_	-0.082	0.041	-0.183	0.675	0.663	0.01	0.054	-0.102	0.02	0.102
	H_e Avg_	0.66	0.50	0.29	0.37	0.11	0.12	0.49	0.42	0.58	0.18
	H_o Avg_	0.70	0.47	0.32	0.17	0.06	0.10	0.48	0.48	0.58	0.18

Sample size, microsatellite variability, in breeding coefficient (F_IS_), Observed and expected heterozygosity for *A. polynesiensis *samples collected in the Society Islands, French Polynesia, 2005-2008. N = sample size, N_all _= number of alleles, F_IS _= the inbreeding coefficient, which is a measure of the reduction of heterozygosity of a subpopulation due to non-random mating, * = significant (*P *< 0.026) after a Bonferroni correction for heterozygote deficit using a Fisher's exact test

**Table 4:** *F*_ST _estimates for all population pair-wise comparisons

	Maupiti	Raiatea	Tahaa	Moorea	Tahiti
**Maupiti**	-	0.104*	0.217*	0.099*	0.102*
**Raiatea**	0.091	-	0.038	0.034*	0.030
**Tahaa**	0.201	0.038	-	0.113*	0.128*
**Moorea**	0.076	0.033	0.113	-	0.020*
**Tahiti**	0.083	0.030	0.131	0.021	-

Samples from multiple collection sites on each island were pooled and combined as one sample for each island. Above diagonal: not corrected for null alleles, below diagonal: corrected for null alleles using ENA [27]. *Indicates significant difference from zero after a Bonferroni correction (*P *< 0.005)

Out of 532 tests for linkage disequilibrium, four were significant after a Bonferroni correction. The four significant tests were between locus AP1 and APT2 in populations, MP, MPA, MOG2, and NF. However, in the remaining populations these loci remained completely unlinked. Hence, we found no evidence of subdivision among populations due to linked loci.

### Temporal genetic differentiation

A low but a significant level of differentiation was suggested by the pair-wise *F*_ST _value (0.041, *P *< 0.05) between samples collected in March and July of 2008 on motu Toamaro. However, since *F*_ST _values were low, samples were not differentiated in further analyses. No evidence of temporal genetic differentiation was suggested by the non-significant pair-wise *F*_ST _values between March and November (0.030), and July and November (0.006) samples. Low and/or non-significant *F*_ST _values suggest little evidence of temporal genetic variation between samples collected at different times at Gump Station, Moorea (-0.015). Since little to no differentiation was observed between temporal samples from motu Toamaro and Gump Station Moorea, all collections were included in the subsequent analyses examining for intra- and inter island differentiation.

### Intra-island genetic differentiation

An AMOVA indicates little difference among populations within islands. Differences between populations within islands accounted for only 4.83% of the variation. However, the variation contributes significantly to analyses (*P *< 0.00001) (Table 5), which may account for the low but significant *F*_ST _values observed from samples from Tahiti and Raiatea (Table 6). In addition, no intra-island differentiation on Tahiti, Raiatea, and Maupiti were suggested by *F*_ST _values (Table 6). To determine if any genetic differentiation on Raiatea was the result of sampling on motus and the main island, samples were pooled from the main island of Raiatea and compared to five motu samples using pair-wise *F*_ST _values. Differentiation was low in all comparisons (*F*_ST _= -0.0078-0.08). As another method to examine for intra-island genetic differentiation, a Bayesian analysis using STRUCTURE was performed. Results indicated sampled populations on Raiatea belonged to one genetic cluster. STRUCTURE was not able to show population substructure between mainland island samples of Raiatea and motu samples as suggested by pair-wise *F*_ST _tests. Separate analyses using STRUCTURE were conducted to examine for population sub-structuring on Maupiti and Tahiti, demonstrating one genetic cluster each for samples from Maupiti and Tahiti.

**Table 5:** Results of an AMOVA testing for genetic structure in *A. polynesiensis *sampled in French Polynesia

	**d.f**.	Sum of Squares	Variance components	% variation	P
Among islands	4	25.4	0.052	5.49	< 0.00001
Among populations within islands	14	30.8	0.046	4.83	< 0.00001
Within populations	557	459.5	0.852	89.68	0.00587

**Table 6:** Intra-island population genetic differentiation

Island group	***F***_**ST**_	Confidence interval
Maupiti	0.026	-0.009 - 0.080
Raiatea	0.036	0.016 - 0.063*
Tahiti	0.065	0.020 - 0.132*

*If the confidence interval does not include zero, then *F*_ST _is significantly different from zero

### Inter-island genetic differentiation

**Figure 2:**
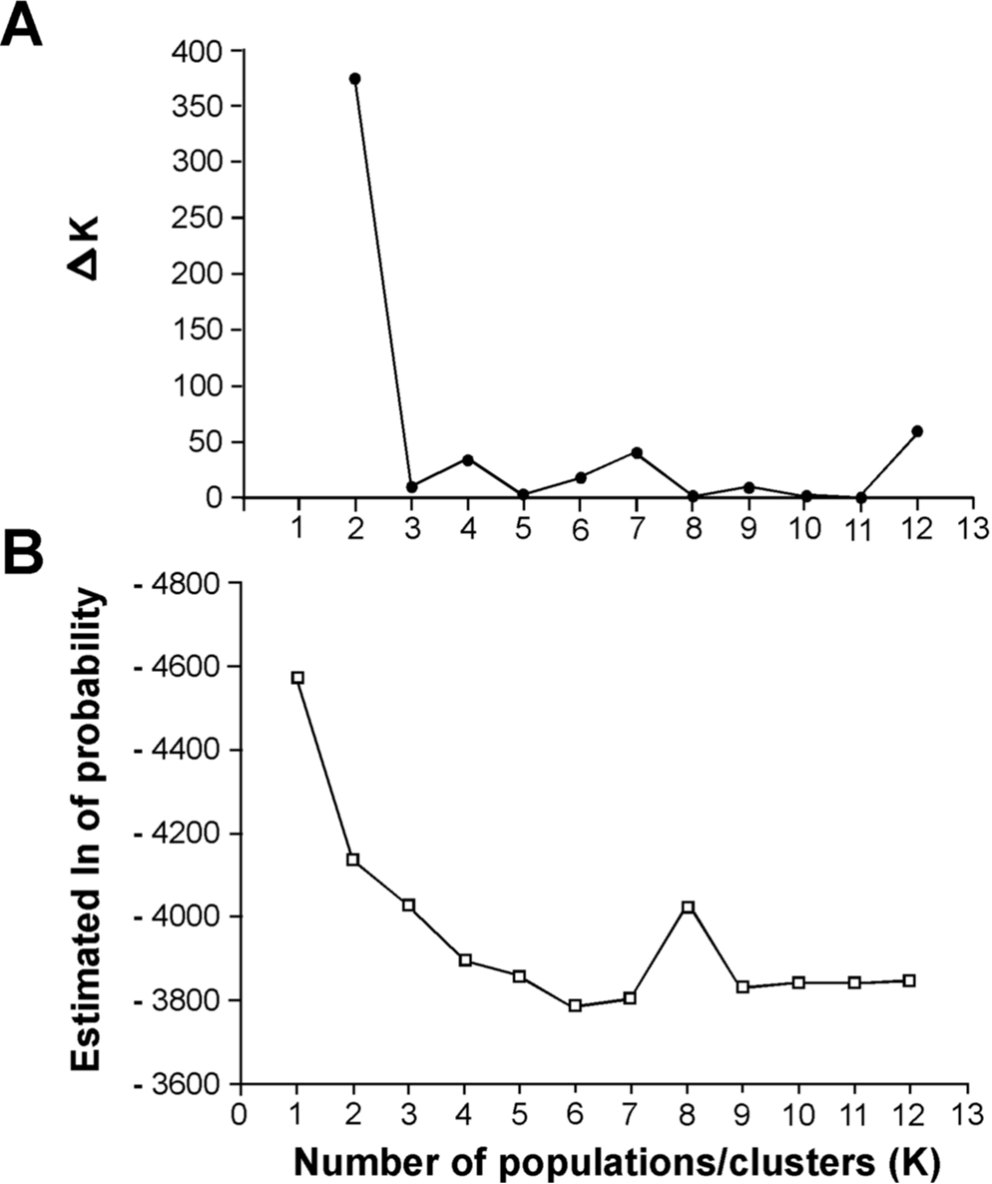
**Neighbor-joining tree base on Nei's standard genetic distance **[32]**for *A. polynesiensis *samples collected in the Society Islands, French Polynesia, 2005-2008**. Scale bar represents genetic distance of 0.01.

Significant pair-wise *F*_ST _values translate to a relatively minor level of genetic differentiation, and suggest Maupiti, Tahaa, and Moorea are differentiated from each other and Tahiti, while Raiatea is not significantly differentiated from Tahaa and Tahiti, but differentiated from Maupiti and Moorea (Table 4). This pattern is also clear from the neighbor-joining tree constructed using Nei's genetic distance values (Figure 2), which suggests a greater genetic distance of Tahaa and Maupiti from the other islands. An AMOVA indicates little difference among islands. Differences between islands accounted for only 5.49% of the observed variation, however, the existing variation while small contributes significantly to the observed genetic differentiation (Table 5).

**Figure 3:**
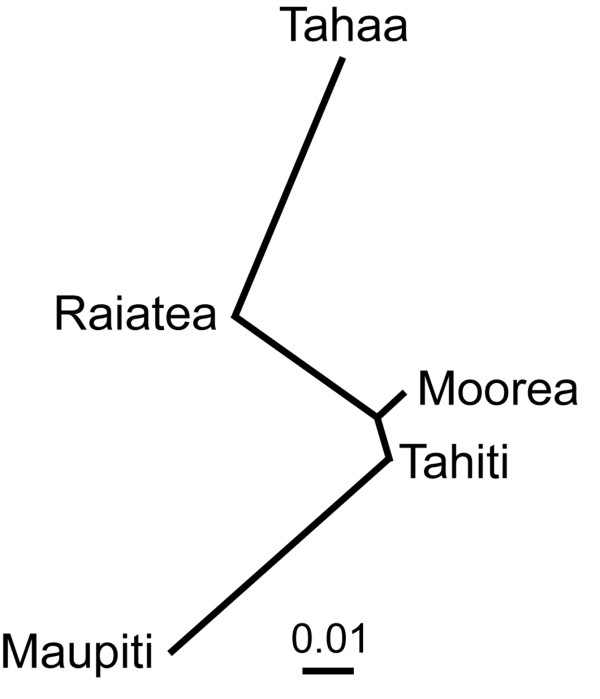
**The estimated number of genetic populations according to STRUCTURE from *A. polynesiensis *samples collected in the Society Islands, 2005-2008**. (A) The rate of change of log-likelihood values (delta K) for estimating the number of genetic populations. The highest value for delta K indicates the number of estimated genetic populations. (B) Log-likelihood values for the estimated number of genetic populations. The highest values associated with a plateau in the graph indicate the most likely number of genetic populations.

As an alternate method to examine for inter-island differentiation and population genetic structure of the Society Islands, a Bayesian analysis of population inference using STRUCTURE was performed (Figure 1). The two methods used to determine the number of clusters (Δ*K *(Figure 3A and ln likelihood of *K *(Figure 3B)) suggest the presence of three clusters based upon the sampled populations (Figure 3). In Figure 1, the probability of each individual belonging to one of the three clusters is presented. STRUCTURE analyses suggest there are three genetic clusters: (1) a Maupiti and the CP lab strain cluster, (2) an admixed population on Raiatea, Moorea, Tahiti, Tahaa, and (3) an AR cluster.

### Isolation by distance

**Figure 4:**
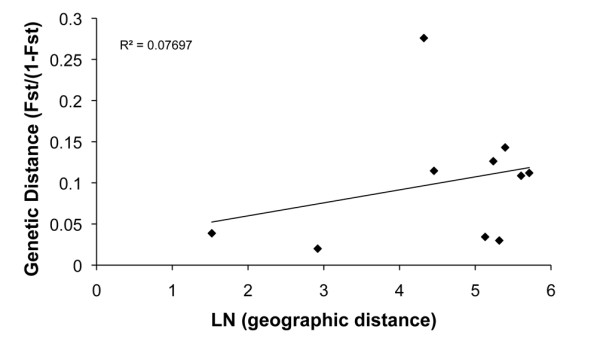
**Linear regression of pair-wise *F*_ST _values of pooled island samples versus geographic distance for *A. polynesiensis *samples collected in French Polynesia, 2005-2008, indicating isolation by distance**.

Pooled samples from each island were used to determine if geographic distance is influencing the observed genetic structure. While there was an increase in *F*_ST _values associated with geographic distance, the correlation was not significant, suggesting geographic distance cannot fully explain the observed pattern of genetic structure (*P *> 0.32) (Figure 4).

### Analysis of mosquito movement

Analyses using BAYESASS, suggests Raiatea and Tahiti are the largest immigration sources to the other island groups (*m >*0.11) (Table 7). However, the direction of movement of mosquitoes is unidirectional from these two islands to Maupiti, Tahaa, and Moorea, with little evidence for immigration from Maupiti, Tahaa, and Moorea (Table 7).

**Table 7:** Migration rate estimates using BAYESASS

	Source				
**Destination**	**Maupiti**	**Raiatea**	**Tahaa**	**Moorea**	**Tahiti**
**Maupiti**	**0.851** (0.720-0.985)	*0.112* (0.002-0.239)	0.004 (2.1^-5^-0.022)	0.016 (1.7^-5^-0.069)	0.017 (7.8^-5^-0.064)
**Raiatea**	0.010 (5.1^-5^-0.035)	**0.836** (0.787-0.877)	0.003 (6.15^-5^-0.011)	0.016 (0.02^-2^-0.069)	*0.135* (0.095-0.179)
**Tahaa**	0.015 (2.63^-5^-0.067)	*0.262* (0.172-0.321)	**0.692** (0.667-0.743)	0.016 (2.45^-5^-0.073)	0.015 (4.73^-5^-0.069)
**Moorea**	0.016 (0.01^-2^-0.067)	*0.131* (0.005-0.274)	0.008 (4.46^-5^-0.031)	**0.695** (0.667-0.777)	*0.152* (0.025-0.262)
**Tahiti**	0.005 (1.87^-5^-0.026)	*0.222* (0.167-0.268)	0.004 (1.05^-5^-0.018)	0.005 (1.28^-5^-0.027)	**0.764** (0.720-0.813)

Values shown are the means of the posterior distributions of *m*, the migration rate (proportion of first generation migrants) into each population, and their respective 95% confidence intervals in parentheses. Migration rates greater than 0.1 are underlined and italicized. Values in bold are the proportion of individuals derived from the source population each generation. Column headings are the source population, and row headings are the destination populations

## Discussion

Our study describes the population genetic structure of medically important *A. polynesiensis *in Society Islands of French Polynesia. Results suggest minor levels of genetic variability of *A. polynesiensis *populations across the Society Islands. It was expected that the structuring of the Society Islands would have resulted in substantial genetic differentiation between different island groups based upon an island model of population differentiation [36]. The observed pattern of population genetic structure may be the result human-assisted movement, of adult mosquitoes and/or eggs. BAYESASS analysis suggests a significant amount of movement occurs between the more populated islands of Moorea, Tahiti, and Raiatea, such that the gene flow counteracts genetic drift and limits the amount of differentiation. Previous studies have suggested a link between the amount of air and maritime traffic and the levels of genetic differentiation of *A. polynesiensis *[11]. Results presented here suggest a similar link. Results are also similar to previous reports observing limited amounts of genetic differentiation of populations of *Aedes taeniorhynchus *among the Galapagos Islands attributed to human assisted mosquito movement of mosquitoes [37]. The islands of Moorea, Tahiti, and Raiatea have the largest populations of inhabitants and substantial air and maritime traffic [38]. Furthermore, Moorea, Tahiti, and Raiatea have developed a substantial tourism industry over the last several decades. Transport of tourists and commodities are probable contributors to mosquito dispersal. Mosquito eggs, which are capable of drying and surviving for weeks are a likely life stage for dispersal. While mosquito flight under their own power is possible across small distances to nearby motu, assistance by wind and storms would be a likely requirement for larger distances between islands. Sampling of populations from additional islands, including more isolated islands would help to better define the role played by air and maritime traffic in the observed pattern of genetic differentiation.

Minor levels of genetic differentiation according to pair-wise *F*_ST _values were observed when comparing Maupiti, Tahaa, and Moorea to the other islands. Tahaa, while geographically close to Raiatea, does not have an airport or large harbor; instead, it is primarily accessed by small boats. Maupiti has an airport, but it is the most geographically isolated of the sampled sites and receives relatively few flights, therefore may be less affected by human assisted movement of mosquitoes. Furthermore, the airport is located on a motu (population not sampled). Thus, based on the source-sink hypothesis, air introductions of mosquitoes to a motu would have a reduced probability of moving to the main island and other motus. Moorea is geographically close to Tahiti and receives a considerable amount of commercial traffic from Tahiti, suggesting considerable mosquito movement between these two islands as suggested by low pair-wise *F*_ST _values (0.020) and significant levels of migration (*m = *0.152) (Table 7). Mosquito movement could also be related to population size. Larger populations of mosquitoes on Tahiti and Raiatea could be providing more emigrants than smaller populations on other islands (e.g., Maupiti, Tahaa, and Moorea) due to their large effective population size.

Results from the STRUCTURE analysis agree with *F*_ST _estimates and suggest low levels of differentiation among islands except for Maupiti, which falls into a separate genetic cluster. Maupiti is the most geographically isolated island supporting the existence of two genetic clusters in the natural population. Log likelihood values also confirm the presence of three clusters two of which exist in the natural population and the other the *A. riversi *laboratory population. The observed clustering pattern also suggests that the CP strain has accumulated an allelic distribution, like that of wild type populations from Maupiti. The observed CP genotype is consistent with the introgression of the *A. riversi *genetic background into the *A. polynesiensis *laboratory strain created from a source on Maupiti [7]. As expected, since AR is a different species, a distinct genetic cluster was formed when compared to the laboratory populations and wild populations. The CP strain is intended for release as part of a field trial to test a *Wolbachia-*based IIT strategy in French Polynesia. The confirmation that the genetic makeup is like that of the indigenous population, especially collections from Maupiti suggests the strain is genetically compatible with natural populations. Furthermore, the CP strain displays high rates of CI with naturally infected populations [7,10], suggesting that the CP strain would be befitting for releases. An analysis of temporal genetic variation of samples from motu Toamaro suggest low but significant differentiation between samples collected in March 2008 and July 2008. Detection of variation between two samples within the same year versus samples collected more than two years apart might be explained by seasonal variation between the populations. Seasonality in French Polynesia is defined as a wet and dry season. The wet season typically lasts from November to April, and the dry season lasts from May to October. Smaller populations on the motu would be more susceptible to seasonal fluctuations and rainfall. As part of a different study, smaller populations were associated with the dry season when compared to the wet season [39]. In smaller populations, the relative importance of genetic drift and rates of inbreeding can increase, resulting in an increase in homozygosity and loss of genetic variation that can impact the observed population genetic structure. In this study, smaller populations in the dry season were shown to have higher rates of homozygosity compared to the wet season. While, comparisons of samples collected only during the dry season were not differentiated. Future studies could include additional sampling to test the hypothesized link between population genetics, size and seasons in the small motu populations.

The observed results also have epidemiological implications. Previous studies have suggested that variation in vector competence can be associated with geographic location [15,18,40]. Furthermore, studies examining the vector competence and population genetics of *A. polynesiensis *from different collection localities throughout the Society Islands may help determine the role of genetic differentiation on vector competence. While little population genetic structure was observed within islands, results suggest the presence of two distinct populations on less inhabited islands of Maupiti and Tahaa. Variation in genotype may be responsible for variation in vector competency, but is yet to be determined. Further experimentation examining the vector competence of different genotypes collected from different localities (e.g., Maupiti and Tahiti) may have implications for the epidemiology of disease transmission and will contribute to the planning of vector control strategies.

## Conclusion

Results presented here are relevant to the design and implementation of area-wide control strategies such as genetic control approaches (e.g., RIDL) [41] and autocidal approaches such as SIT [42,43] or a *Wolbachia*-based IIT strategy [7,44-46]. An additional *Wolbachia-*based strategy is focused on population replacement, where the reproductive advantage afforded by *Wolbachia *induced CI as a population replacement strategy to drive wanted phenotypes into natural populations [44,47]. Understanding the patterns of gene flow and population genetic structure of *A. polynesiensis *is important for the design and interpreting the outcome of area-wide elimination techniques. If the genotype of released individuals is different than natural populations, emigrants and immigrants out of targeted and into control populations can be identified, respectively. These findings also highlight the importance of obtaining baseline population genetic data prior to area-wide control strategies to understand potential re-infestation events after such a strategy. Populations identified as genetic sinks may be better suited as initial release areas, minimizing the risk of released mosquitoes establishing in untargeted areas for IIT and replacement strategies. Data suggests that motu surrounding Maupiti are potential sites for initial releases of cytoplasmically incompatible males as part of an IIT or replacement strategy, since there is little evidence of emigration and immigration to and from the main island.

## Abbreviations

ITS2: Internal transcribed spacer 2; CI: Cytoplasmic incompatibility; CP: Hybrid B-type *Wolbachia *infected strain (described in [7]); AMOVA: Analysis of Molecular Variance; AR: *Aedes riversi *University of Kentucky laboratory colony; HWE: Hardy-Weinberg equilibrium; RIDL: Release of insects carrying dominant lethal; SIT: Sterile insect technique; IIT: Incompatible insect technique; CI: Cytoplasmic incompatibility

## Competing interests

The authors declare that they have no competing interests.

## Authors' contributions

CLB and SLD conceived and designed the experiments. CLB performed the experiments. CLB analyzed the data. CLB and SLD wrote the paper and both authors approved the final version of the manuscript.
